# Design of EM Sensor for Non-Contact Detection of Defective Wire Harness in Conveyor System

**DOI:** 10.3390/s22197350

**Published:** 2022-09-28

**Authors:** Hailin Zeng, Jeong-Hyun Park, Mincheol Kim, Jae-Gon Lee

**Affiliations:** 1 Department of Convergence IT Engineering, Kyungnam University, Changwon 51767, Korea; 2 Department of Electronic Engineering, Kyungnam University, Changwon 51767, Korea

**Keywords:** electromagnetic (EM) sensor, microstrip patch antenna, non-contact detection, dual-feed, wire harness

## Abstract

For a non-contact detection of defective wire harness in conveyor system, a new method using the electromagnetic (EM) sensor is proposed in this paper. A dual-feed and multi array microstrip patch antenna operating at 5.8 GHz is utilized to design the EM sensor. When the wire harness is located above patch antenna, the equivalent circuit of each patch antenna and wire harness can be modeled as shunt resistor, capacitor, and inductor. Moreover, a capacitive coupling between the patch antenna and the wire harness is generated. Next, the shunt resistor of wire harness increases due to the defect of the wire so that the reflection coefficient of the patch antenna is lower than that of the wire without defect; thus, the defect of wire harness can be detected by magnitude of reflection coefficient at resonant frequency. The performances of the designed EM sensor are verified and compared by the equivalent circuit modeling, full-wave simulation, and measurement.

## 1. Introduction

Generally, the EM sensor that operate at the strongest radio frequency response is non-destructive evaluation technology and it is widely used in various applications such as medical biosensor, damage detection for infrastructures, and so on [1,2,3,4,5,6,7,8,9,10,11,12,13,14,15,16]. In [1], a fluidic glucose sensor operated at 2.4 GHz using dual complementary split ring resonators (CSRRs) with a switching circuit is proposed for non-invasive and continuous glucose concentration detection. Structural diagnostic techniques using EM waves also have been used to determine the condition of a structure [2].

Wire harnesses, which is grouping wires to keep the wires neat, orderly, and hidden from view, are utilized to provide power and electrical signals to electrical and electronic devices inside various electrical equipment such as the vehicle, computer, and ship. To detect defects in the wire harness, most of the wire harness companies have adopted a direct contact method to check a flow of current. However, the direct contact method may have some problems in mass production. Lots of connectors in the detection system for each type of wire harness are required to measure the amount of the current. In addition, it takes time to connect the wire harness and the connector in the detection system by hand, and the signal line in the connector may be distorted in the process. To overcome the problems of the direct contact method, the new type of non-contact detection of defective wire harness in conveyor system is proposed and investigated using the EM sensor in this paper. A planar type of microstrip patch antenna is employed for the EM sensor. When the defect of the wire is placed above patch antenna, the reflection coefficient of the patch antenna is different from that of the non-defect wire due to the change of inducing current in the wire. After obtaining the reflection coefficients of various wire harnesses without defect, the defective wire harness can be found based on the reference data. In Section 2, the operation principle of a designed EM sensor including an equivalent circuit model is described, and the dual-feed and the multi array EM sensors that can detect defect regardless of the orientation and the position of the wire harness on the conveyor belt are also investigated. The measured results for real wire harnesses are discussed to verify feasibility of proposed EM sensor in Section 3. Finally, the conclusions are presented in Section 4.

## 2. EM Sensor for Non-Contact Detection of Defective Wire Harness

**Figure 1:**
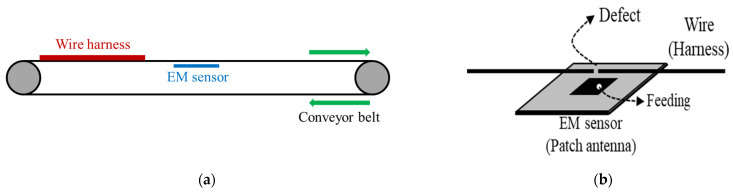
EM sensor for non-contact detection of defective wire harness. (**a**) Conveyor system. (**b**) Wire harness above the EM sensor.

The EM sensor for non-contact detection of defective wire harness in conveyor system is represented by Figure 1. When the wire harness with a defect is moved by conveyor system and the defect is above the EM sensor, the reflection coefficient of the patch antenna is lower than that of the non-defect wire harness. Considering the reflection coefficient of the patch antenna, it thus can be determined that there is a defect in the wire without direct contact between the wire and the detection system. A microstrip patch antenna is employed to design the EM sensor in this paper. The patch antenna is designed as a rectangular patch with a length 18 mm of, a width of 16 mm, and a ground size of 30 mm to operate at 5.8 GHz. A coaxial feeding is utilized and is 4.5 mm away from center. Furthermore, the substrate for the patch antenna is RT/duroid 5880 with a relative permittivity of 2.2, a thickness of 1.6 mm, and a loss tangent of 0.0009. To verify the feasibility of a non-contact detection, the dimensions of a utilized wire are as follows. The diameter and the length of a wire are 1 mm and 400 mm, respectively. The length of a wire defect is 1 mm, and the spacing between the wire and the patch antenna is 5 mm.

**Figure 2:**
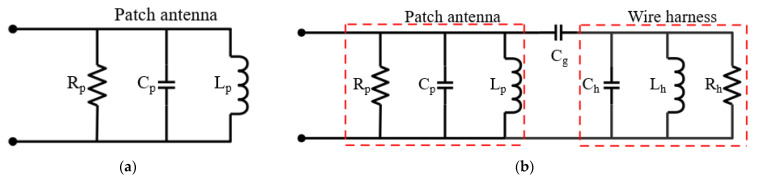
Equivalent circuit model. (**a**) Patch antenna. (**b**) Wire located above patch antenna.

**Figure 3:**
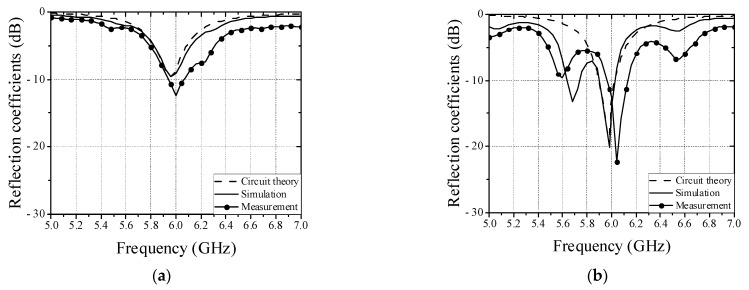
Comparison of reflection coefficients among equivalent circuit model, full-wave simulation, and measurement. (**a**) Non-defect wire. (**b**) Defective wire.

Figure 2 shows the equivalent circuit model of a patch antenna and a wire located above patch antenna. The patch antenna can be modeled as a parallel resistance (*R_p_*), capacitance (*C_p_*), and inductance (*L_p_*). Since the wire also can be modeled as a parallel resistance (*R_h_*), capacitance (*C_h_*), and inductance (*L_h_*), and a capacitance (*C_g_*) can be generated between the patch antenna and the wire, the equivalent circuit model of the wire above patch antenna is represented by Figure 2b. To produce capacitive coupling between the patch antenna and the wire at dominant mode, the wire must be positioned in the direction of the feeding point and center of patch antenna, as shown in Figure 1b. The circuit parameters of the patch antenna with operation frequency of 5.8 GHz in the Figure 2a were obtained with *R_p_* = 57.7 Ω, *C_p_* = 8.96 pF, and *L_p_* = 0.084 nH. The circuit parameters of non-defect wire are as follows: *C_h_* = 10.08 pF, *L_h_* = 0.068 nH, and *R_h_* = 43 Ω. In addition, the circuit parameters of defective wire are as follows: *C_h_* = 6.8 pF, *L_h_* = 0.097 nH, and *R_h_* = 103.8 Ω. Since the induced current from the patch antenna decreases due to the defect, the shunt resistor (*R_h_*) of the defective wire increases compared to that of non-defect wire. Next, the reflection coefficient of the patch antenna is lower than that of the non-defect wire, as shown in Figure 3. The utilized simulator and vector network analyzer (VNA) are commercial ANSYS Electronics desktop software (2021) and Anritsu MS46522B, respectively. The compared results show excellent agreement except for an additional parasitic resonance by the interaction between patch antenna and wire. The measured reflection coefficients of patch antenna at the resonant frequency in cases of non-defect and defective wires are about −12.4 dB and −22.4 dB, respectively.

**Figure 4:**
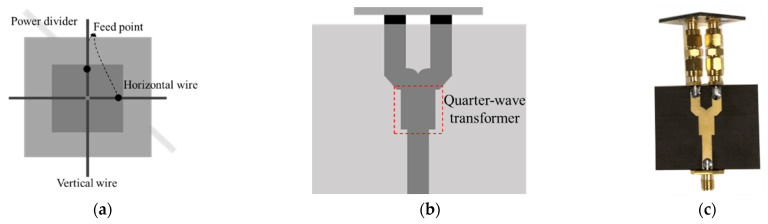
Structure of dual-feed patch antenna (**a**) Top view. (**b**) Side view. (**c**) Fabricated dual-feed patch antenna.

**Figure 5:**
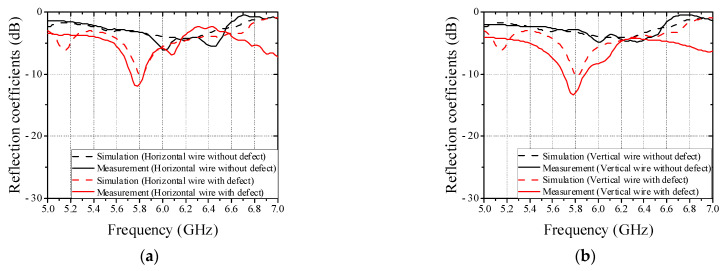
Full-wave simulated and measured reflection coefficients of patch antenna in cases of wires without and with defect. (**a**) Horizontal wire. (**b**) Vertical wire.

When the direction of the wire harness coincided with the center line at the feeding point, the difference in the magnitude of the reflection coefficients of wire harness with and without defect is maximized. As results of full-wave simulation, the rotation angle at which the difference of more than 5 dB is 60 degrees. To detect regardless of the orientation of the wire harness above conveyor belt, the dual-feed patch antenna for EM sensor is employed as shown in Figure 4. The patch antenna operating at 5.8 GHz is designed as a rectangular patch with a length 16.7 mm of and a width of 16.7 mm. Each coaxial feeding is positioned 7 mm away from center and a quarter-wave transformer is utilized to match two transmission lines with different impedances. Figure 5 shows the full-wave simulated and measured reflection coefficients of patch antenna against horizontal and vertical wires in cases of wires without and with defect. The differences of reflection coefficients of patch antenna at the resonant frequency are measured to be 8.8 dB and 10.4 dB in cases of horizontal and vertical wires, respectively.

**Figure 6:**
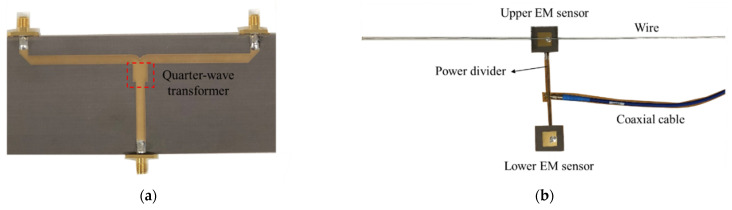
Structure of multi array EM sensor. (**a**) Power divider. (**b**) Dual EM sensor.

**Figure 7:**
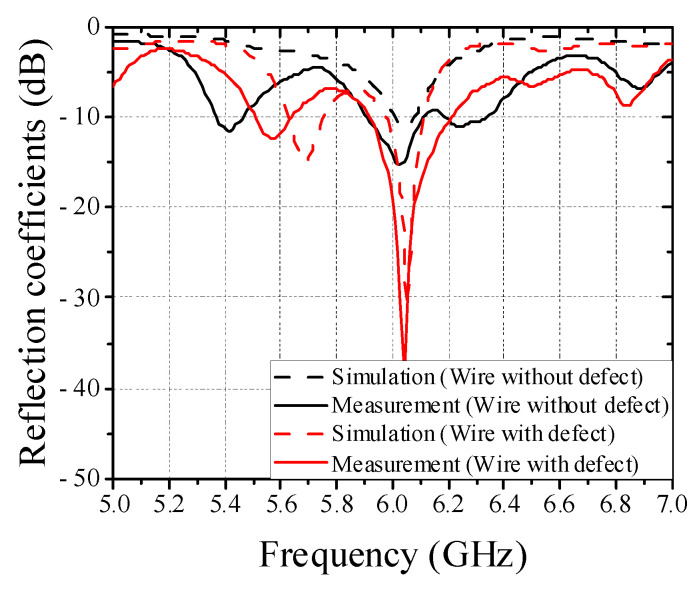
Full-wave simulated and measured reflection coefficients of upper patch antenna in cases of wires without and with defect.

Moreover, the multi array EM sensors are needed to detect defect regardless of the position of the wire harness on the conveyor belt. To confirm the feasibility, we have designed dual EM sensor using a power divider with quarter-wave transformer, as shown in Figure 6. Figure 7 shows the full-wave simulated and measured reflection coefficients of upper patch antenna in cases of wires without and with defect. When one wire harness with a defect is moved by conveyor system, the differences of reflection coefficients of patch antenna at the resonant frequency are measured to be about 20 dB for the upper and lower sensors. Thus, it is possible to detect whether a wire harness with a defect moves above upper or lower sensor from the simulated and measured results.

## 3. Measured Results for Real Wire Harness

**Figure 8:**
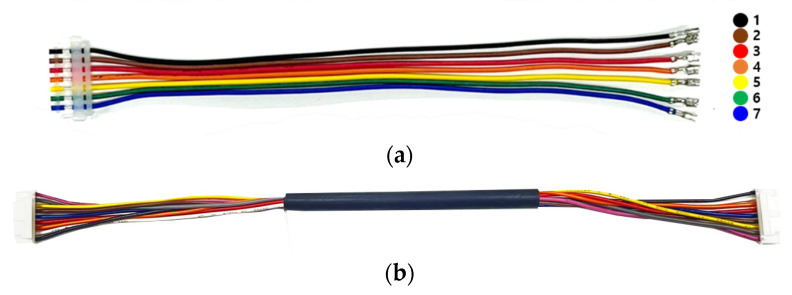
Photograph of real harness. (**a**) Flat harness with 7 wires. (**b**) Circular harness with 9 wires.

**Figure 9:**
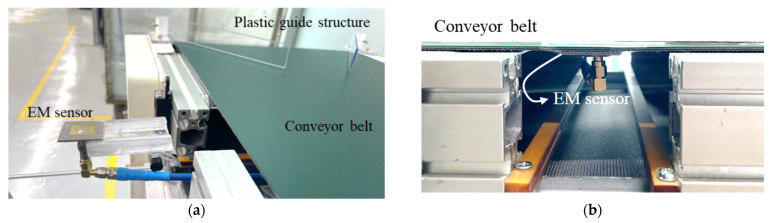
Photograph of experiment setup including EM sensor. (**a**) Before mounting below conveyor belt. (**b**) After mounting below conveyor belt.

**Figure 10:**
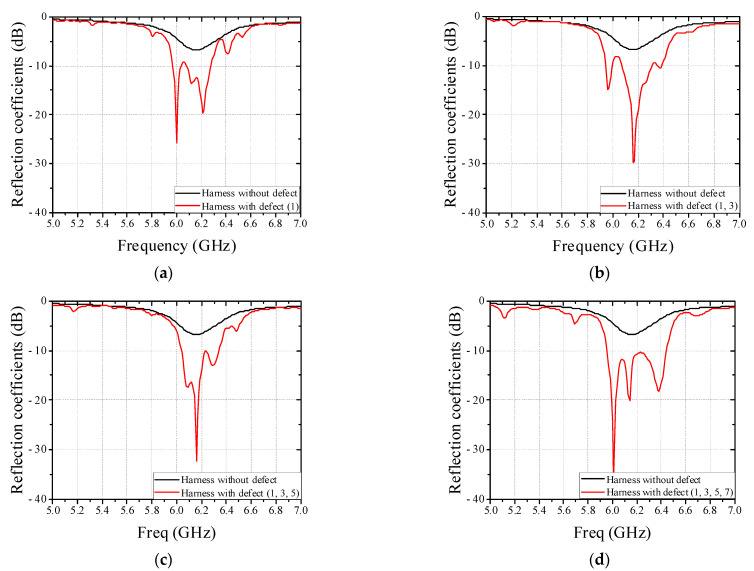
Measured reflection coefficients of patch antenna in cases of real flat harness (7 wires) without and with defect according to defect location. (**a**) Defective wire: 1. (**b**) Defective wire: 1 and 3. (**c**) Defective wire: 1, 3, and 5. (**d**) Defective wire: 1, 3, 5, and 7.

**Figure 11:**
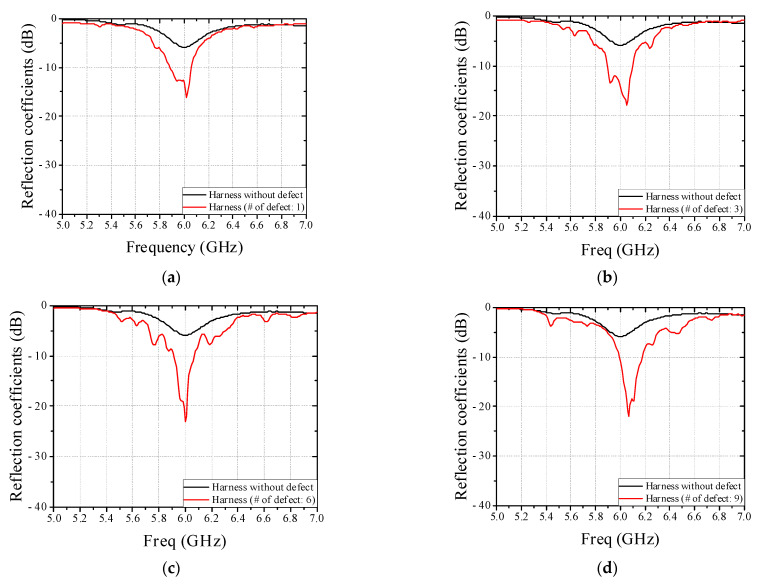
Measured reflection coefficients of patch antenna in cases of real circular harness (9 wires) without and with defect according to the number of defective wire. (**a**) The number of defective wire: 1. (**b**) The number of defective wire: 3. (**c**) The number of defective wire: 6. (**d**) The number of defective wire: 9.

To verify feasibility of non-contact detection method of defective wire harness in conveyor system based on results in Section 2, the real flat harness with 7 wires and circular harness with 9 wires are utilized and measured in this paper, as shown in Figure 8. Figure 9 shows a conveyor system with EM sensor. The EM sensor is mounted on a jig and can be controlled the position under the conveyor belt in a slide type. Since the real harness experiment is performed using a single EM sensor, a plastic guide structure is employed to pass the harness through the same location, as shown in Figure 9a. Furthermore, the change of the reflection coefficient was confirmed by operating the conveyor belt. The harness defect is artificially created in the center and the length of a wire harness defect is 1 mm. We have simulated the parameter study against the length of a wire defect and obtained the detection limit of 0.1 mm. Figure 10 shows the measured reflection coefficients of patch antenna in cases of real flat harness (7 wires) without and with defect according to defect location. In addition, Figure 11 shows the measured reflection coefficients of patch antenna in cases of real circular harness (9 wires) without and with defect according to the number of defect wire. The measured results were obtained when a defect of harness passed over the EM sensor. The maximum differences of reflection coefficients of patch antenna at the resonant frequency were measured to be more than 10 dB in all cases. It is necessary to display the number of defective wire harness on the monitor, in order to utilize the proposed EM sensor in a smart factory. When the threshold value is set at the resonant frequency band and the reflection coefficient value below the threshold value is measured, it is recognized as a defective harness. Thus, if difference of reflection coefficients of patch antenna at the resonant frequency is large, the defect detection rate increases. It is concluded that a non-contact detection methodology of defective wire harness in conveyor system using the EM sensor is valid.

## 4. Conclusions

A non-contact detection methodology of defective wire harness in conveyor system using the EM sensor is proposed and investigated in this paper. Microstrip patch antenna operating at 5.8 GHz is utilized to implement the EM sensor. A difference between wire harness without and with defect in reflection coefficient of the patch antenna occurs so that the defect of wire harness can be detected. Moreover, the dual-feed and the multi array EM sensors that can detect defect regardless of the orientation and the position of the wire on the conveyor belt are also investigated. Finally, from experimental results of real wire harness, it is concluded that novel non-contact detection methodology of defective wire harness in conveyor system using the EM sensor has good performances and the proposed detection system would be one of solutions for a detection of defective wire harness.

## Data Availability

Not applicable.
